# Cycloheximide promotes paraptosis induced by inhibition of cyclophilins in glioblastoma multiforme

**DOI:** 10.1038/cddis.2017.217

**Published:** 2017-05-18

**Authors:** Lin Wang, Justin H Gundelach, Richard J Bram

**Affiliations:** 1 Department of Biochemistry and Molecular Biology, Mayo Clinic, Rochester, MN, USA; 2 Department of Immunology, Mayo Clinic, Rochester, MN, USA; 3 Department of Pediatric and Adolescent Medicine, Mayo Clinic, Rochester, MN, USA

## Abstract

Cancer is the second leading cause of death worldwide. Current treatment strategies based on multi-agent chemotherapy and/or radiation regimens have improved overall survival in some cases. However, resistance to apoptosis often develops in cancer cells, and its occurrence is thought to contribute to treatment failure. Non-apoptotic cell death mechanisms have become of great interest, therefore, in hopes that they would bypass tumor cell resistance. Glioblastoma multiforme (GBM), a grade IV astrocytic tumor is the most frequent brain tumor in adults, and has a high rate of mortality. We report that NIM811, a small molecule cyclophilin-binding inhibitor, induces catastrophic vacuolization and cell death in GBM cells. These unique features are distinct from many known cell death pathways, and are associated with an incompletely defined cell death mechanism known as paraptosis. We found that NIM811-induced paraptosis is due to unresolved ER stress. The abnormal upregulation of protein translation was responsible for the build-up of misfolded or unfolded proteins in ER, whereas pro-survival autophagy and UPR signals were shutdown during prolonged treatment with NIM811. Although cycloheximide has been claimed to suppress paraptosis, instead we find that it only temporarily delayed vacuole formation, but actually enhanced paraptotic cell death in the long term. On the other hand, mTOR inhibitors rescued cells from NIM811-induced paraptosis by sustaining autophagy and the UPR, while specifically restraining cap-dependent translation. These findings not only provide new insights into the mechanisms underlying paraptosis, but also shed light on a potential approach to enhance GBM treatment.

Glioblastoma multiforme (GBM) is the most malignant brain tumor and is essentially always associated with a poor prognosis.^1^ Common treatments, including surgical resection, radiation or chemotherapy, are primarily given without the expectation of permanent cure, and can only offer patients a median survival rate of about 1 year.^2^ Therefore, it is crucial to develop novel therapies that could improve survival.

GBM cells frequently develop genetic alterations that help them escape the apoptotic programmed cell death pathway.^3^ Hence, discovery and utilization of new cell death mechanisms could lead to improved GBM treatments. Paraptosis, an atypical cell death pathway, was first described in 2000.^4^ It has loosely been characterized by morphologic criteria (including ballooning cytoplasmic vacuolization and retention of normal nuclear architecture) and non-involvement of the caspases.^5^ Inhibition by the protein synthesis inhibitor cycloheximide has also been a key feature.^5, 6, 7^

Cyclophilins have long been known as foldases and protein chaperones.^8^ Recently, cyclophilins have been found to be overexpressed in many different types of cancers, and have been thought to support malignant transformation,^9^ by enhancing survival of cancer cells. Small molecule inhibitors of the cyclophilins may therefore be useful as anticancer agents. Although cyclosporine A has a high binding affinity to the majority of cyclophilins,^10^ it is not a suitable drug for the treatment of cancer because of its toxic effects on the kidneys and the immune system.^11^ Specifically, the cyclosporine A-cyclophilin complex leads to inhibition of calcineurin, a serine/threonine protein phosphatase that is required for NF-AT-mediated T-cell activation.^12^ We have instead explored the potential of using NIM811 (MeIle4-cyclosporine), which is a cyclosporine derivative that does not interfere with T-cell activation because it lacks affinity for calcineurin,^13^ and has higher binding affinity for the peptidyl-prolyl isomerase (PPIase) domain of cyclophilins.^14^

Here we used NIM811 to induce death in GBM cells, and found that its primary mode of action is to induce paraptosis. By deciphering the cellular events that take place after NIM811 treatment, we have obtained an in-depth understanding of the cell transition points from life to death during this process. We were also able to identify the critical pathways required for GBM cell survival. Importantly, we found that cycloheximide could only provide a brief inhibition of vacuolization but unexpectedly facilitated cell death in the long run. Our findings indicate that NIM811 activates paraptosis through promoting protein translation and simultaneously hindering the UPR response and autophagy activity, which together lead to irreversible disruption of the ER and cell death.

## Results

### NIM811-induced paraptosis-like cell death in GBM cells

**Figure 1:**
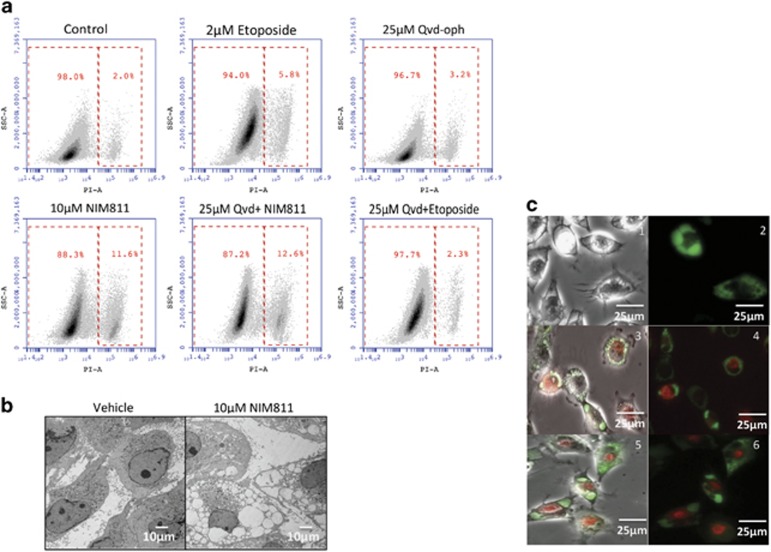
NIM811 induces ER vacuolization and paraptosis cell death in GBM cells. (**a**) Pan-caspase inhibitor Qvd-oph was not able to rescue cell death induced by 10 *μ*M NIM811 at 24 h. (**b**) Electron microscopic images revealing the morphological structures of vacuoles, scale bar=10 *μ*m. (**c**) Labeling cells with GFP-ER signal sequence of calreticulin-KDEL (1–2) and TagRFP-Leader sequence of E1 alpha pyruvate dehydrogenase (3-4) or RFP-lamp1 (5-6) indicate that the vacuoles originate from the ER. Scale bar=25 *μ*m

Based on the observations that cyclophilins are upregulated in many types of cancers,^15^ especially in brain tumors,^16^ we tested the ability of NIM811 to kill several human GBM cell lines. Treating the cell lines, U251 and T98G, with NIM811 indeed caused substantial degrees of cell death when used at 10–15 *μ*M concentrations after approximately 24 h (Supplementary Figures 1A and 1B). Unexpectedly, rather than the typical morphological changes characteristic of apoptosis, we found that NIM811 induced the formation of massive vacuoles inside of the cells before death (Supplementary Figures 1B and 13). Unlike etoposide or staurosporine-initiated apoptosis, NIM811-treated cells did not go through an early stage in which they would stain for Annexin-V by virtue of phosphatidyl-serine exposure and negative for propidium iodide (PI).^17, 18^ Instead, NIM811-treated cells transitioned directly from PI-, Annexin-V double-negative to double-positive stained dead cells (Supplementary Figures 1C and 11). Furthermore, addition of the pan-caspase inhibitor Qvd-oph^19^ did not rescue death elicited by NIM811, although did rescue etoposide or staurosporine-induced apoptosis (Figure 1a and Supplementary Figure 10). Knocking down of Apaf1 or MLKL was also incapable of blocking NIM811-induced death (Supplementary Figure 12). The necroptosis inhibitor Necrostatin-1 (ref. 20) was effective at suppressing shikonin-stimulated^21^ killing, but was unable to save NIM811-treated cells (data not shown). Electron microscopic examination revealed the inside structure of NIM811-treated U251 cells, in which large single membrane-bound vacuoles surrounded the intact nuclei (Figure 1b). Together, these observations suggested that NIM811 may induce death via paraptosis.^5^

Previous reports have indicated that paraptotic death is accompanied by formation of vacuoles that arise from either the ER or the mitochondria.^6, 7^ To identify the source of vacuoles in NIM811-treated cells, we transfected them with an expression construct that fuses GFP with the ER signal sequence and KDEL ER retention domain of calreticulin to enable long-term labeling of ER. After 20 h of NIM811 treatment, the vacuoles colocalized precisely with the ER-GFP fluorescence. However, co-transfecting with mitochondria or lysosome labeling constructs showed no colocalization, indicating that the vacuoles were exclusively generated from ER (Figure 1c).

### NIM811 delays tumor growth in mice

**Figure 2:**
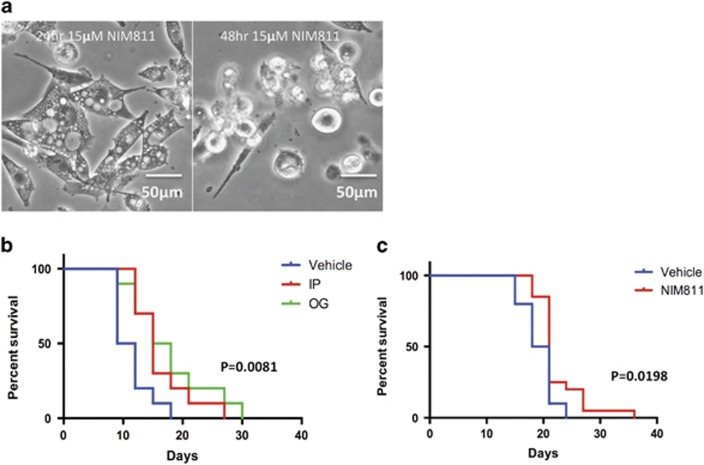
NIM811 inhibits tumor growth *in vivo* (**a**) 15 *μ*M of NIM811 caused vacuolization in G22VF cells at 24 h, and substantial cell death at 48 h. Scale bar=50 *μ*m. (**b**) Nude mice (*n*=30) were injected subcutaneously with 2E^6^ G22VF cells. When the mice developed tumors larger than 100 mm^3^, they were randomly divided into three groups to receive treatments: vehicle (1% ethanol+9% cremophor EL+ 90% normal saline), NIM811 intraperitoneal (25 mg/kg), NIM811 oral gavage (25 mg/kg). Using 1 cm^3^ as cut-off, NIM811-treated mice had slower tumor growth rate than the mice in vehicle group. Curves were significant different by the log-rank (Mantel–Cox) test, *P*=0.0081. (**c**) Using tumor size of 2.5 cm^3^ as killing criteria, NIM811-treated mice survived longer than vehicle-treated mice. Curves were significantly different by log-rank (Mantel–Cox) test, *P*=0.0198

To determine whether tumor cells may also be subject to NIM811-induced death *in vivo*, we conducted a study using a xenograft model of human GBM. We first verified that human GBM G22VF cells from the Mayo Brain Tumor SPORE core facility behaved similarly to U251 and T98G cells. NIM811 at 15 *μ*M stimulated similar vacuolization of G22VF cells by 24 h and significant cell death at 48 h (Figure 2a). G22VF cells were then subcutaneously inoculated into nude mice (*n*=30). When tumor size reached 100 mm^3^, mice were randomly divided into three groups to receive three times per week of drug treatments: group one received vehicle treatment; group two mice received 25 mg/kg of NIM811 by intraperitoneal injection (IP); group three mice received 25 mg/kg of NIM811 via oral gavage. Although not curative on its own at this dosing schedule, we observed that both cohorts of NIM811-treated mice reached the interim analysis threshold size (1 cm^3^) and mandatory killing size (2.5 cm^3^) significantly later than vehicle only treated mice (Figures 2b and c). We conclude that NIM811 has activity against GBM cells *in vitro* and *in vivo*.

### NIM811-induced vacuolization depends upon protein synthesis but does not require loss of proteasome activity

**Figure 3:**
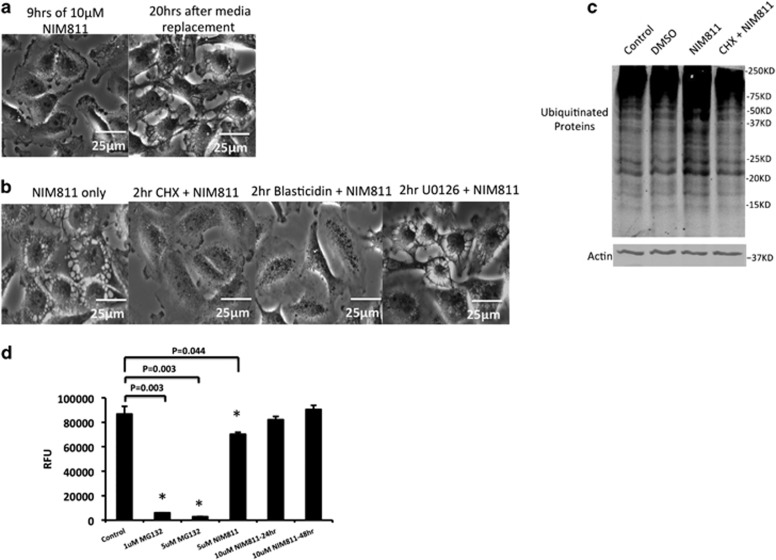
NIM811-induced vacuolization is sensitive to protein synthesis inhibition. (**a**) U251 cells were incubated with 10 *μ*M NIM811 containing media for 9 h, then media were replaced with fresh DMEM (10% FBS). Cells were then tracked by live microscopy (scale bar=25 *μ*m) for 24 h. (**b**) U251 cells were pretreated with 20 *μ*M cycloheximide (CHX) or 10 *μ*g/ml of blasticidin or 20 *μ*M of U0126 respectively for 2 h followed by 24 h 10 *μ*M NIM811 incubation (scale bar=25 *μ*m). (**c**) Twenty hours of NIM811 treatment leads to accumulation of ubiquitinated proteins, pretreatment with 20 *μ*M cycloheximide for 2 h prevented this build-up. (**d**) After 24 h or 48 h of 10 *μ*M NIM811 treatment, cells were collected for 20S proteasome activity assay. Cells treated with 1 or 5 *μ*M MG132 served as positive control for this assay

Although NIM811 treatment typically took at least 20 h to cause vacuolization and cell death, we found that these effects were irreversibly initiated by as little as 9 h of exposure to the compound (Figure 3a). Previous studies on paraptosis have suggested that vacuolization and death may depend upon ongoing protein synthesis and inhibition of proteasomal activity, thus leading to ER stress.^5, 6, 7^ To determine the impact of NIM811 on these functions, we first explored the role of protein synthesis using inhibitors. As was shown in the case of other paraptotic inducers, cells exposed to cycloheximide for only 2 h before treatment with NIM811 did not develop cytoplasmic vacuoles, indicating that the brief interruption of protein synthesis prevented the formation of enlarged ER structures 48 h later. We also tested blasticidin, a completely different protein synthesis inhibitor,^22^ and found that it also was able to prevent NIM811-induced vacuolization (Figure 3b). Unlike some inducers of paraptosis, the effects of NIM811 did not require MEK activation,^5, 7, 23^ as U0126 did not block vacuolization (Figure 3b).

Paraptosis has been associated with accumulation of misfolded or unfolded proteins,^23^ so we examined total proteins in NIM811-treated cells for ubiquitination, which targets polypeptides for proteasome-mediated degradation.^24^ Indeed, overnight incubation of NIM811 greatly augmented the amount of ubiquitinated proteins, an effect that was alleviated by cycloheximide pretreatment (Figure 3c). Accumulation of ubiquitinated proteins could indicate that NIM811 suppresses proteasomal activity. However, NIM811-treated cells had almost normal activity of proteasomes even after prolonged treatment (Figure 3d).

### NIM811 elicited early activation of ER stress, autophagy and mTOR signaling

**Figure 4:**
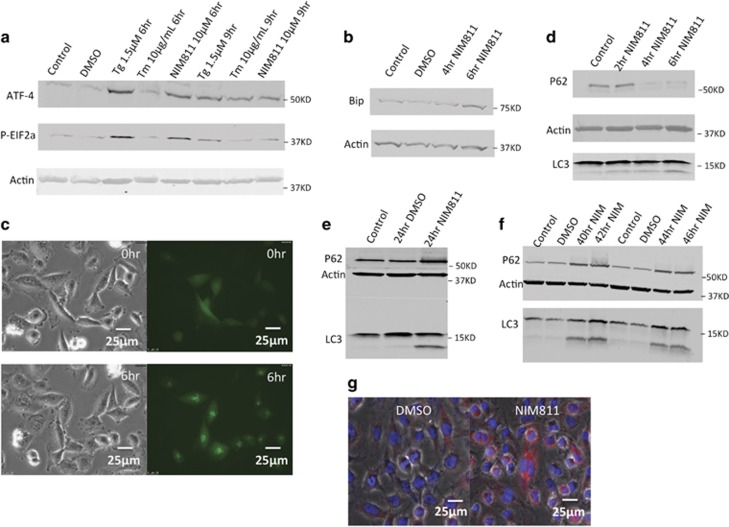
NIM811 induced a transient upregulation of UPR and autophagy. (**a** and **b**) In all, 0 *μ*M NIM811 transiently induced UPR signaling (P-EIF2a, Bip, ATF4, CHOP upregulation) at 6 h, but except for Bip, the activation of UPR mediators disappeared at 9 h. Tunicamycin (Tm) and thapsigargin (Tg) were used as positive controls for the UPR. (**c**) In total, 10 *μ*M NIM811 was added to GFP-LC3B transfected U251 cells and imaged by fluorescence microscopy at 0 h and 6 h. Scale bar=25 *μ*m. LC3 puncta were detected at 3–6 h. LC3 puncta formation occurred before the appearance of vacuoles. (**d**) LC3-I and -II conversion occurred at 4–6 h of 10 *μ*M NIM811 treatment accompanied by p62 degradation. (**e** and **f**) In all, 10 *μ*M NIM811 incubation caused p62 accumulation at 24 h, which persisted at later time points (40–46 h). (**g**) Immunofluorescence staining of SQSTM1/p62 (red) and nucleus (blue) after 24 h vehicle (left) or 10 *μ*M NIM811-treated (right) U251 cells. Scale bar=25 *μ*m

In order to identify potential mechanisms underlying NIM811-associated cell death, we performed RNA-seq to compare gene expression profiles between NIM811-treated cells and cells treated with vehicle. As anticipated from the marked impact of NIM811 on ER morphology, we found upregulation of multiple ER stress response genes, including HSPA5, ATF3, ATF4 and CHOP (Supplementary Table 1). Based on western blotting, ATF4 and P-eIF2a increased transiently during early exposure to NIM811 (Figure 4a). BIP was induced to higher levels, which were maintained for 48 h, together consistent with prolonged ER stress (Figures 4b and 7d) and with transient activation of the UPR.

Unexpectedly, the gene encoding ATG8 was significantly elevated, and several other genes related to autophagy including LC3B, p62, ULK1 were also upregulated in NIM811-treated cells (Supplementary Table 1). Induction of autophagy in NIM811-treated cells was verified by transfecting U251 cells with GFP-tagged LC3B.^25^ We observed the formation of LC3-positive puncta starting shortly after addition of drug, and becoming more extensive by 3–6 h of exposure to NIM811 incubation, long before the appearance of cytosolic vacuoles (Figure 4c). Western blotting for LC3-I to II conversion and p62 degradation also verified activation of autophagy at approximately 4–6 h of treatment (Figure 4d). However, by 24 h, p62 protein loss was reversed, and it began to accumulate, regardless of continued LC3-I to -II conversion (Figures 4e and g) suggesting that autophagy activity may be impaired after prolonged NIM811 treatment.

**Figure 5:**
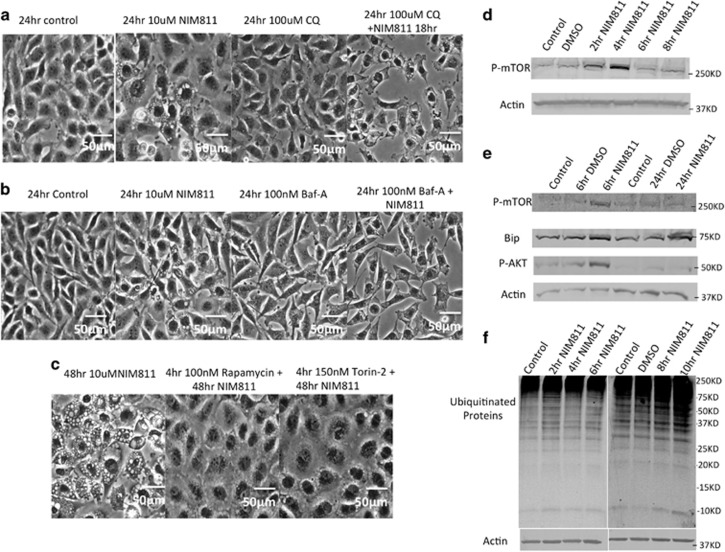
mTOR inhibitors block NIM811-induced vacuolization. (**a** and **b**) U251 cells were treated with 10 *μ*M NIM811 in combination with 100 *μ*M chloroquine (CQ) or 100 nM bafilomycin A1 (Baf-A) for 24 h. Scale bar=50 *μ*m. (**c**) Four-hour pretreatment with 100 nM rapamycin or 150 nM torin-2 blocked vacuolization in U251 cells at 48 h. Scale bar=50 *μ*m. (**d** and **e**) Western blotting of NIM81-treated cells demonstrated transient stimulation of mTOR as well as AKT phosphorylation at 2-6 h. Bip remained upregulated after 24 h. (**f**) Cell lysates were collected from 2 h to 10 h of 10 *μ*M NIM811-treated U251 cells, and analyzed for protein ubiquitination

Increased rates of autophagy may be an effect of NIM811 by which it induced death, or may arise in response to cellular stress, to resist death. To determine the relationship between autophagy and cell death, we combined NIM811 with the autophagy inhibitors chloroquine^26^ or bafilomycin-A1^27^ then monitored vacuole formation and survival. Chloroquine and bafilomycin A1 accelerated NIM811-induced vacuolization and death (Figures 5a and b), and suppressed Bip expression (Supplementary Figures 8A and 8B), suggesting that autophagy may be a survival response in these cells. In addition, shRNA-mediated knockdown of Beclin-1 or ATG5 made U251 cells more sensitive to NIM811-induced paraptotic effects (Supplementary Figures 8C and 8E). Conversely, pretreatment of cells with the mTOR inhibitors rapamycin^28^ or torin-2^29^ to activate autophagy significantly decreased vacuolization (Figure 5c), further supporting the idea that the induction of autophagy in response to NIM811 resists or delays the ultimate cellular demise.

Given the significant effect of the mTOR inhibitors, we examined its state in NIM811-treated cells. Importantly, we observed a substantial activation of both mTOR and its potential upstream activator AKT, as evidenced by the appearance of phospho-mTOR and phospho-AKT, which peaked between 2 and 6 h after addition of NIM811 (Figures 5d and e). This activation preceded the accumulation of unfolded ubiquitinated proteins that began 10 h from NIM811 addition (Figure 5f). A 4-h pretreatment with either mTOR inhibitor diminished the accumulation of ubiquitinated proteins in NIM811-treated cells (Supplementary Figure 2A), and converted the upregulation of P-eIF2a from a transient to a sustained course, lasting over 48 h (Supplementary Figure 2B).

### NIM811 stimulates both Cap-independent (CI) and Cap-dependent (CD) translation

Together, these data implicated cell vacuolization and death as arising from uncontrolled delivery of proteins into the ER in the face of impaired or insufficient handling of these misfolded proteins, perhaps because of failure to fully activate autophagy and failure to sustain activation of the UPR. In this case, suppressing protein synthesis by cycloheximide or blasticidin blocked the development of ER-derived vacuoles. On the other hand, mTOR inhibition suppressed vacuole formation most likely through increasing autophagy and extending the time course of the UPR. However, mTOR itself is known to regulate several components involved in protein synthesis.^30^

**Figure 6:**
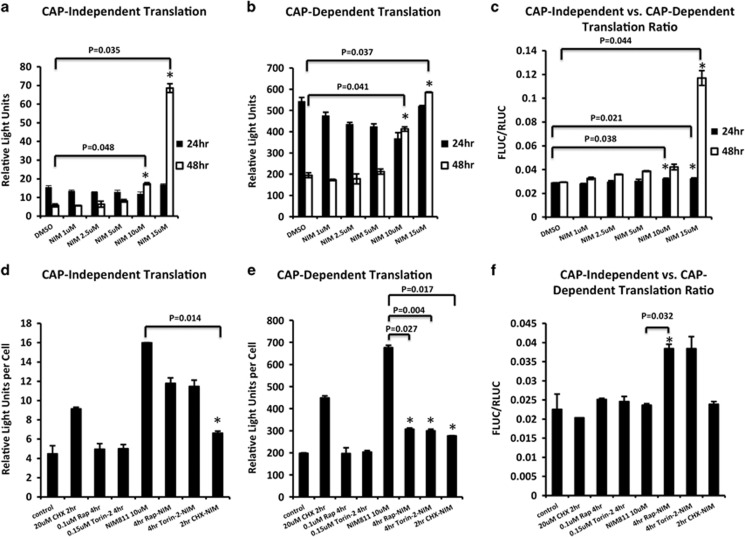
NIM811 activates both CD and CI translation. (**a-c**) CD and CI translation were measured by the dual renilla/firefly luciferase assay after 24 or 48 h of indicated treatments, and relative light units were normalized to cell numbers. NIM811 significantly increased the CI and CD translation at 10 and 15 *μ*M, *P<0.05. (**d-f**) Pretreatment of rapamycin, torin-2 effectively decreased CD translation during NIM811 incubation, whereas brief pretreatment with cycloheximide substantially decreased both CD and CI translation. *P<0.05

Two types of protein translation systems exist in cells, having opposing roles in ER stress.^31, 32, 33^ Typically, under conditions of ER stress, the UPR activates PERK, which phosphorylates eIF2a.^34^ P-eIF2a specifically downregulates CD translation but spares CI translation to alleviate the overall protein burden in the ER and increase the synthesis of ER chaperones, respectively.^35, 36, 37^ This allows the ER increased time and capacity to process incoming proteins. We transfected cells with a bicistronic luciferase plasmid^38^ to help differentiate CD translation from CI translation under the influence of various concentrations of NIM811 at 24 and 48 h of incubation. Both CD and CI translations were increased with increasing concentrations of NIM811 at 48 h (Figures 6a and c). Interestingly, the inappropriate acceleration of CD translation at 48 h occurred most robustly at NIM811 concentrations that corresponded to those that caused cell vacuolization and death (10 and 15 *μ*M).

We next addressed the question as to whether short treatments with rapamycin, torin-2 or cycloheximide would impact the two branches of protein translation at late time points after NIM811 addition. U251 cells were pretreated for 2 or 4 h with the inhibitors, and then replaced with NIM811 containing media for 48 additional hours before testing using the dual-luciferase assay. Short treatments with either of the mTOR inhibitors or with cycloheximide restrained the abnormal CD translation induced by NIM811 at 48 h (Figure 6e). However, cycloheximide was unique in also suppressing CI translation, whereas the mTOR inhibitors allowed increased CI translation to continue (Figure 6d). Importantly, the ratio of CI to CD translation was significantly enhanced by brief pretreatment with rapamycin; in agreement with its ability to suppress vacuolization in NIM811-treated cells (Figure 6f). However, it was puzzling that cycloheximide treatment also inhibited vacuolization yet did not induce the favorable change in this ratio, thus raising the question of how it could allow survival in the face of NIM811 treatment.

**Figure 7:**
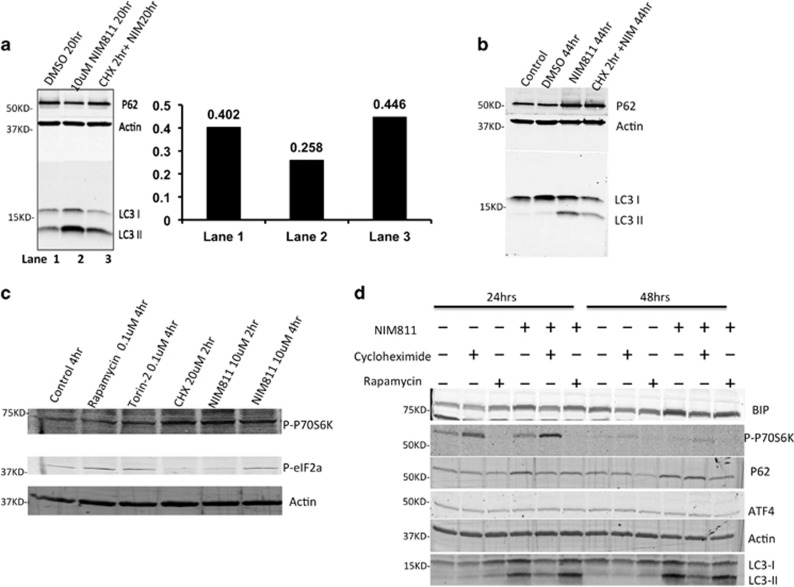
Cycloheximide inhibits autophagy and UPR signaling, whereas activating downstream mTOR substrate phosphorylation. (**a**) Brief treatment with cycloheximide led to increased p62 accumulation at 20 h of NIM811 treatment (quantitation with the actin loading control on the right). (**b**) Two-hour cycloheximide pretreatment decreased LC3-I and II levels even after prolonged NIM811 treatment (44 h), (**c**) 10 *μ*M NIM811 stimulated phosphorylation of P70S6K at 2 h and p-EIF2a at 4 h, while 2 h of cycloheximide increased the P-P70S6K level but decreased eIF2a phosphorylation. (**d**) Two-hour cycloheximide pre-incubation boosted the p-p70S6K level after 24 h, whereas, with rapamycin pretreatment, P70S6K phosphorylation decreased and p62 was degraded more efficiently

To try to resolve this, we performed RNA-seq on total mRNA from cells pretreated with cycloheximide for 2 h, and then incubated with NIM811 for 20 h. Comparison with NIM811 alone-treated cells, RNA-seq revealed significant downregulations in genes associated with autophagy (ATG8, ULK1 and P62) and UPR (ATF4 and ATF3) by cycloheximide (Supplementary Table 2). In agreement with this, cycloheximide partially suppressed the NIM811-induced conversion of LC3-I to II, and early loss of p62, suggesting that cycloheximide suppressed, rather than enhanced autophagy, unlike the mTOR inhibitors (Figures 7a and b). Furthermore, cycloheximide decreased NIM811-induced P-EIF2a levels, but instead activated phosphorylation of the mTOR target P70S6K, which would serve to exacerbate the protein overload coming into the ER (Figure 7c). At 24 h, cycloheximide still enforced the continued strong induction of P70S6K phosphorylation, whereas suppressing LC3 conversion and Bip expression (Figure 7d). These contrasted with effects of rapamycin, which increased P62 clearance and reduced the levels of P-P70S6K.

**Figure 8:**
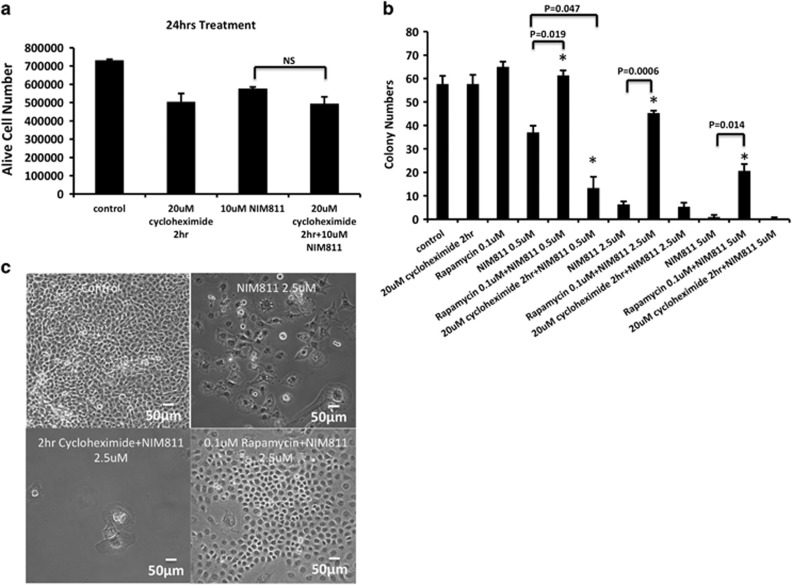
Cycloheximide pretreatment exacerbates cell death induced by prolonged NIM811 treatment. (**a**) Cycloheximide pretreatment did not improve U251 live cell numbers after 24 h of 10 *μ*M NIM811 incubation. (**b**) Colony number counts on day 13 indicates that pretreatment with rapamycin significantly increased colony numbers over NIM811 alone treatment (from 0.5 to 5 *μ*M), *P<0.05, whereas, cycloheximide pretreatment substantially decreased colony numbers by 0.5 *μ*M NIM811 incubation, *P<0.05. (**c**) Microscopic images were obtained on day 13 to demonstrating the morphology of the colony cells. Rapamycin also blocked the vacuolization triggered by NIM811. Scale bar=50 *μ*m

Although the ability of cycloheximide to reverse paraptotic vacuolization and cell death has been described multiple times, its effects on the UPR, autophagy, and CI to CD ratio, which differed significantly from those of mTOR inhibitors, raised the question of whether vacuolization should truly be equated with cell death. To reveal this connection between cell death and vacuolization, we compared the cell numbers between NIM811 only treated cells and the ones pretreated with 2 h cycloheximide. Shockingly, although cycloheximide could efficiently delay vacuole formation, it failed to provide any survival benefit to cells at 24 h of culture (Figure 8a), nor at earlier time points (Supplementary Figure 9). To more accurately assess cell survival, we used colony formation assays. Two hours of treatment with cycloheximide alone had no impact on colony formation. However, brief pretreatment with cycloheximide markedly enhanced the amount of cell death caused by NIM811 (Figures 8b, c and Supplementary Figure 3). Conversely, rapamycin significantly reversed the effects of NIM811 (Figures 8b, c and Supplementary Figure 3). Similar to rapamycin, torin-2 substantially restored the colony numbers and reduced vacuolization within NIM811-treated cells (Supplementary Figures 4A and 4C).

### NIM811-induced paraptosis is cyclophilin dependent

To determine whether NIM811 mediates paraptosis depends on its function as cyclophilin inhibitor, we applied shRNA mediated depletion to knockdown two major human cyclophilins: cyclophilin A and B.^15^ Interestingly, a modest degree of spontaneous cytoplasmic vacuolization was initiated after the loss of cyclophilin A or B (Supplementary Figures 7A and 7B), and moreover, the knockdown cells became paraptotic in response to NIM811 treatment at much lower concentrations. All cyclophilin A and B depleted cells formed cytoplasmic vacuoles within 12 h of 2.5 *μ*M NIM811 incubation, which was not observed in control knockdown cells (Supplementary Figure 7C). Thus, we conclude that NIM811 induces paraptosis by blocking the anti-paraptotic effects of these cyclophilins.

## Discussion

Here, we have identified NIM811 as a novel inducer of paraptotic cell death in GBM cells, with the signature finding of ballooning cytoplasmic vacuoles that displace the morphologically unchanged nucleus. NIM811-induced vacuoles arose from the ER, and there was evidence for prolonged activation of ER stress. Several findings underlined the importance of the complex time course of signaling events during the early (reversible) and late (irreversible) stages of NIM811-induced cell death. The early response was accompanied by three transient events: induction of the UPR, acceleration of autophagy and mTOR activation. These occurred before the irreversible stage, which began around 10 h, and which featured accumulation of unfolded ubiquitinated proteins, enlargement of cytosolic vacuoles to accommodate them, and ultimately, increased protein synthesis (Supplementary Figures 5A and 5C). Our favored model is that mTOR activation and increased protein synthesis contributed to the ultimate cell death, whereas transient activation of autophagy and the UPR resisted cell death unsuccessfully.

Although the precise target of NIM811 is not clarified yet, we suspect that its ability to bind and inhibit many of the cellular cyclophilins underlies its activity. In addition to blocking the action of CypB and CypC in the ER,^39, 40^ it is also able to bind to the CypD in the mitochondria and the primary cytosolic CypA.^40, 41^ Depleting cyclophilin A and B accelerated NIM811-induced cellular vacuolization, thus further supporting the idea that NIM811 mediates paraptosis in a cyclophilin-dependent manner.

Importantly, NIM811 significantly boosted both CD and CI protein synthesis. The accepted model for relief of ER stress is that CI proteins are primarily chaperones or other foldases that assist with the business of processing incoming CD proteins.^32, 33^ Thus, the ratio of CI to CD translation should increase in response to a heightened state of ER stress. Although 10 *μ*M of NIM811-induced evidence of ER stress, the UPR was aborted, and the ratio of CI/CD did not change from that of untreated cells. However, addition of an mTOR inhibitor appropriately increased the CI/CD ratio and dramatically reversed vacuolization and suppressed cell death.

The effects of the protein synthesis inhibitor cycloheximide on NIM811-induced paraptosis events deserve additional study in the future. On the one hand, its addition completely blocked the development of cytosolic vacuoles, as has been shown for other paraptotic drugs. On the other hand, although others have claimed that cycloheximide also prevents paraptotic death, we found the opposite that it causes enhanced cell death when combined with NIM811. We suspect an important cause of this was its negative impact on both CD and CI protein translations as well as autophagy. As a result, the CI/CD ratio did not improve from untreated or NIM811 alone treated cells, and ER stress would not have been effectively resolved. Its suppression on autophagy further sensitized the cells to death. In re-examining published reports of paraptosis, we find that others did not typically perform colony-forming assays or FACS analysis, and instead primarily evaluated cell death within a short time period (1 day or so) of drug treatment. Therefore, it is possible that others missed the additive or synergistic effects of interruption of protein synthesis on paraptotic death in their systems. Thus, we carefully tested cycloheximide pretreatment either in other cell lines followed by NIM811 treatment (Supplementary Figure 9), or with two other paraptosis inducers ophiobolin A^6^ and celastrol^7^ at both 24 and 72 h. We found that cycloheximide only prevented vacuolization but failed to save cells from death (Supplementary Figure 6). This result further indicates that ER vacuolization may be a protective response generated by cells to isolate defective proteins within the ER in order to prevent those misfolded aggregates to damage the cellular homeostasis. For that reason, simply inhibiting the vacuole formation without clearing the ER protein build-up would not be sufficient to terminate the cell death.

This result also underlines the importance of evaluating cellular responses in non-apoptotic cell death experiments over multiple time frames to judge the true impact. After all, the essential goal is to eradicate malignant cells over the course of weeks to years, rather than over 24 h.

A striking finding from this work is the marked and long-lasting impact of a 2–4 h pretreatment of cells with inhibitors of either protein synthesis or mTOR. Particularly in the latter case, rapamycin or torin-2 exposure for as little as 4 h was sufficient to allow essentially normal cell proliferation up to 2 weeks later. This effect was accompanied by a change in the ER stress response, most clearly demonstrated by the prolonged phosphorylation of eIF2a, which is known to increase the CI/CD ratio. We hypothesize that this reveals the presence of a competitive race, beginning at the onset of drug exposure, between effective inductions of ER stress resistance via the UPR *versus* stress-induced loss of effective UPR signaling resulting from impaired ER chaperone function. Although still hypothetical, we note that cyclophilin B has been shown to associate in complexes with Bip, CHOP and Grp94,^42, 43^ all of which have known roles in mediating the UPR response. Furthermore, we previously showed that knockdown of cyclophilin B could impair certain aspects of the UPR.^16^ As ER stress intensifies, therefore, the functions of the UPR components may become impaired, and so become unable to transmit a 'distress' signal, thus leading to a worsening spiral of stress-induced destruction. On the other hand, a brief delay in the ER stress induced by rapamycin or torin-2, via enhanced autophagy and elevation of the CI/CD ratio, may be sufficient to allow the UPR to stay ahead of the NIM811-induced stress.

## Materials and methods

### Cell culture and reagents

GBM cell lines (U251 and T98G) were grown in DMEM, 10% FBS. G22VF cells were generated as previously described,^44^ and were also grown in the same media as U251 cells. Reagents (cycloheximide, blasticidin, Qvd-oph, chloroquine, tunicamycin and thapsigargin) were purchased from Sigma-Aldrich (St. Louis, MO, USA). U0126 and bafilomycin-A1 were purchased from InvivoGen (San Diego, CA, USA). NIM811 was requested from Novartis (Basel, Switzerland). Rapamycin was from Alfa Aesar (Ward Hill, MA, USA), and torin-2 was obtained from Tocris Bioscience (Bristol, UK).

### Plasmid transfection and cellular organelle labeling

pEGFP-LC3 (human) was a gift from Toren Finkel (Plasmid #24920, Addgene, Cambridge, MA, USA), and pcDNA3 RLUC POLIRES FLUC was a gift from Nahum Sonenberg (Plasmid #45642, Addgene), ER, mitochondria and lysosome were labeled with cellLight reagents (C10590, C10601 and C10597, Thermo Fisher Scientific, Waltham, MA, USA).

### 20S proteasome activity assay

Cell lysate triplicates were harvested after indicated treatments. Then, the proteasome activities were measured by BML-AK740 assay kit from Enzo life sciences (Farmingdale, NY, USA).

### Western blot

Cell lysates were prepared at indicated time points, and the procedures were described elsewhere.^45^ Antibodies used were the followings: mouse anti-ubiquitin (13-1600, Invitrogen, Camarillo, CA, USA), mouse actin (mab1501, EMD Millipore, Billerica, MA, USA), ATF4 (sc-200, Santa Cruz Biotechnology, Dallas, TX, USA), EIF2a (#9722, Cell Signaling Technology, Danvers, MA, USA), P-EIF2a (#9721, Cell Signaling Technology), Bip (ab21685, Abcam, Cambridge, UK), p62 (ab194129, Abcam), LC3B (NB100-2220, Novus Biologicals, Littleton, CO, USA), P-mTOR (#2971, Cell Signaling Technology), P-AKT (#9271, Cell Signaling Technology) and P-P70S6K (#9205, Cell Signaling Technology).

### Immunofluorescence staining

U251 cells were plated on glass slides in six-well plate at a concentration of 0.3E+06 cells per well. After 24 h of DMSO or 10 *μ*M NIM811 treatment, cells were first fixed with 4% formaldehyde (in 1 × PBS), and permeabilized with 0.1% Triton X-100. Then cells were incubated in blocking solution (5% goat serum in 1 × PBS) for 1 h, and stained by primary antibody P62 (Cell Signalling; #7695) for 1 h. Finally, cells were labeled with anti-rabbit IgG (Alexa Fluor 647) for 1 h, and Hoechst 33342 (Thermo Fisher Scientific; 62249) was applied at 1 *μ*g/ml for 3 min. Thereafter, cells were imaged under fluorescence microscope.

### Dual-luciferase assay

On day 0, 5 *μ*g of pcDNA3 RLUC POLIRES FLUC plasmid was transfected into U251 cells that seeded on 10 cm dish. On day 1, cells were splited and seeded 0.3E+06 cells per well onto six-well plates. On day 2, cells were treated with indicated drugs either for 24 or 48 h.

Translations were measured by Dual-Luciferase reporter assay system (E1910, Promega, Madison, WI, USA).

### Cell viability, cell death, colony formation assay

Cell viability test was conducted with PrestoBlue (Thermo Fisher Scientific; A13261) according to the manufacturer's instructions. Live cell numbers were assessed through flow cytometry. For colony formation assay, 100 cells were seeded onto each well of six-well plate on day 0 and treated with drugs as indicated, and colony numbers were quantified by staining with coomassie blue after 13 days of treatments.

### Xenograft experiment

2E^6^ G22VF cells per mice were inoculated to 30 nude mice via subcutaneously injection. When the tumor sizes reached 100 mm^3^, the mice were randomly divided into three groups to receive corresponding treatments three times per week. NIM811 were prepared by dissolving 50 mg of NIM811 into 10 ml of vehicle (0.9 ml of cremophor EL+0.1 ml of ethanol+9 ml of normal saline).

### Statistical analysis

Data were analyzed by two-tailed Student’s *t*-tests to determine the significance of a difference deviation between two means, and significant difference was represented by *P<0.05.

## Supplementary Material

Supplementary Figure 4

Supplementary Figure 4A

Supplementary Figure 4B

Supplementary Figure 4C

Supplementary Figure 5A

Supplementary Figure 5B

Supplementary Figure 5C

Supplementary Figure 6A

Supplementary Figure 6B

Supplementary Figure 7

Supplementary Figure 8A-8D

Supplementary Figure 8E
