# Supplementary material for: Cycloheximide promotes paraptosis induced by inhibition of cyclophilins in glioblastoma multiforme
Source: Cell Death Dis. 2017 May 18;8(5):e2807–. doi: 10.1038/cddis.2017.217 (PMC5520731; doi:10.1038/cddis.2017.217)
Supplement: Supplementary Information [file cddis2017217x1.docx]

**Supplementary Materials and Methods**

**Cell Culture and Reagents**

Pancreatic carcinoma cell line PANC-1, human lung carcinoma cell line A549 and Brest Cancer cell line MDA-MB231were cultured in DMEM with 10% FBS.

**Short hairpin RNA–mediated silencing**

U251cells were transduced overnight with virus generated from TRC1.5 lentiviral short hairpin RNA (shRNA) vectors (Sigma), followed by puromycin selection 24 hours later. Specific sequences are listed below.

TRC 1.5 library

No-target control shRNA (5’-CAACAAGATGAAGAGCACCAA-3’)

CypA shRNA #1 (5’-GTTTGCAGACAAGGTCCCAAA-3’)

CypA shRNA #2 (5’-GTTCCTGCTTTCACAGAATTA-3’)

CypB shRNA #1 (5’-CCGGGTGATCTTTGGTCTCTT-3’)

CypB shRNA #2(5’-TTCACCAGGGGAGATGGCACA-3”)

Beclin-1 shRNA #1(5’-CCCGTGGAATGGAATGAGATT-3’)

Beclin-1 shRNA #2(5’- CCGACTTGTTCCTTACGGAAA-3’)

ATG5 shRNA #1 (5’- GATTCATGGAATTGAGCCAAT-3’)

ATG5 shRNA #2 (5’- CCTGAACAGAATCATCCTTAA-3’)

**SiRNA Transfection**

Dharmacon SMARTpool ON-TARGET plus products (L-003456-01-0005, L-005326-00-0005, D-001810-10-05) were applied to deplete genes: Apaf1 and MLKL.

**RNAseq**

Total RNA was collected from two independent experiments of U251 cells treated under three different conditions: 1) 20hrs of DMSO, 2) 20hrs of 10μM NIM811, 3) 2hrs of 20μM cycloheximide then 20hrs of 10μM NIM811. The samples were then submitted to Mayo Clinic Core Sequencing for gene expression analysis

**Western Blot**

Cell lysates were prepared at indicated time points, and the procedures were described elsewhere.^45^ Antibodies used were the followings: ATG5 (cell signaling # 12994S), Beclin-1 (cell signalling #3495), cyclophilin A (cell signalling #2175), cyclophilin B (Thermo Fisher #PA1-027A), Apaf1 (cell signalling #5088S)

MLKL (cell signalling # 14993)

**Cell viability, cell death, colony formation assay**

Cell death was measured after 24 or 48hrs by FACS analysis of Annexin V/PI staining.

**Supplementary Figure Legends**

**Supplementary Figure 1**: NIM811 induces non-apoptotic cell death in GBM cells (A) U251 cells were treated with DMSO or different concentrations of NIM811 (1μM, 2μM, 4μM, 6μM, 8μM, 10μM, 15μM, 20μM) for 24hrs, followed by cell viability measurement by presto blue assay (B) 24hrs of 10μM NIM811 incubation caused dramatic vacuolization in U251 and T98G cells, scale bar=25μm (C) Annexin V and PI stain illustrated that unlike etoposide, there was no appearance of an early apoptotic population in U251 cells after 48hrs of 10μM NIM811 treatment

**Supplementary Figure 2**: mTOR inhibitors alleviate protein aggregates re-stimulate UPR signalling (S2A) U251 cells were pre-treated with 4hrs 100nM rapamycin or 150nM torin-2 and then followed by 48hrs of 10μM NIM811 treatment. Brief treatment with rapamycin or torin-2 suppressed the ubiquitinated protein accumulation caused by NIM811. (S2B) pre-treating U251 cells with 4hrs of rapamycin or torin-2 helped sustain EIF2a phosphorylation after 24hrs and 48hrs of NIM811 treatments

**Supplementary Figure 3**: (S3) colony formation results at day 13. Cells were treated as indicated.

**Supplementary Figure 4:** Rapamycin and torin-2 efficiently blocked vacuolization induced by NIM811 (S4A-S4B) colony formation assay results at day 13. Both rapamycin and torin-2 pre-treatment were able to decrease the loss of colony numbers caused by NIM811 toxicity, *p<0.05. (S4C) Microscopic images were obtained on day 13 to demonstrating the morphology of the colony cells. Addition of Rapamycin and torin-2 also blocked the vacuolization triggered by NIM811. Scale bar=50μm.

**Supplementary Figure 5**: Proposed model of NIM811 induced cellular death. (S5A) NIM811 induced two stages of cell damage: an early stage (reversible) and a late stage (irreversible). During the reversible stage (0-8hrs), NIM811 activated the mTOR pathway, including the phosphorylation of mTOR and its downstream effector P70S6K, which would serve to accelerate protein production. Perhaps in response to this sudden increase in protein synthesis, mTOR signalling was suppressed, in turn activating autophagy to accelerate the clearance of proteins. The unfolded protein response was also initiated to alleviate stress in the ER. Throughout this stage, cells could be rescued by simply removing the NIM811 containing media. However, in the continued presence of NIM811, cells then entered into a stage of irreversible damage and eventually cell death. During the irreversible stage (after 9hrs), autophagy was overloaded and the UPR was also attenuated, which were followed by the accumulation of ubiquitinated proteins. In order to maintain homeostasis of the ER, misfolded or unfolded proteins were apparently isolated into specific compartments, seen as ER vacuolization. Prolonged NIM811 incubation also triggered increased protein translation, further increasing the ER burden. Unresolved ER stress then led to inevitable cell death. (S5B) With a 2hr cycloheximide pre-treatment, the P-p70S6K level was even more elevated, and the autophagy and UPR pathways were down-regulated. Although cycloheximide decreased protein translation, due to its non-selective suppression of both cap-dependent and cap-independent translations, ER stress was still exacerbated. (S5C) mTOR inhibitors (rapamycin, torin-2) prevented this chain of events by increasing autophagy and activating the UPR response, and by slowing down cap-dependent translation, thus providing survival benefits to cells.

**Supplemental Figure 6**: Cycloheximide inhibits paraptosis induced vacuolization but fails to decrease cell death (S6A) U251 cells were treated with 0.6μM ophiobolin-A (opa) or 0.8μM celastrol with or without 2hrs of 20μM cycloheximide pre-treatment. Microscopic images were obtained at 24hr and 72hr. Scale bar=50μm. (S6B) Live cell numbers under different treatments were measured by flow cytometry at 24hrs and 72hrs. The addition of cycloheximide did not improve live cell numbers by ophiobolin-A or celastrol treatment at 24hrs or 72hrs.

**Supplemental Figure 7**: Cyclophilin A and B knockdown cells are more sensitive to NIM811 treatment (S7A) U251 cells transduced with shCypA and shCypB were collected for western blot to verify the knockdown efficiency (S7B) cyclophilin A or B ablation led to spontaneous vacuolization in U251 cells. Scale bar=50μm (S7C) cyclophilin A or B knockdown cells underwent cytoplasmic vacuolization at much lower and shorter times of NIM811 treatment. Scale bar=50μm.

**Supplemental Figure 8:** Autophagy inhibition facilitates NIM811 induced paraptosis (S8A-S8B) Bafilomycin-A and Chloroquine combination with NIM811 treatment suppressed Bip expression both at early time points (6hr and 9hr) and late time points (24hr and 48hr). (S8C-8D) U251 cells transduced with shATG5 or shBeclin-1 were harvested for western blot to verify the knockdown efficiency. (S8E) ATG5 and Beclin-1 knockdown cells are more susceptible to NIM811 induced paraptosis. Scale bar=50μm

**Supplemental Figure 9:** Cycloheximide promotes NIM811-induced paraptosis in U251, T98G and PANC-1 cells at multiple time points. (S9A) 2hr 20μM cycloheximide pretreatment further decreased live cell numbers under NIM811 treatment at 8hr and 16hr in U251 cells. (S9B-9C) pre-incubating T98G cells with 20μM cycloheximide for 1hr did not affect NIM811 induced paraptosis at 12hr and 24hr, but it caused more cell death at 48hr. (S9D) cycloheximide pretreatment promoted NIM811-mediated death in PANC-1 cells at 24hr and 48hr.

**Supplemental Figure 10:** Pan-caspase inhibitor Qvd-oph fails to rescue T98G and U251 cells from NIM811 mediated paraptosis (S10A-10C) In T98G cels, Qvd-oph successfully inhibited apoptosis that activated by staurosporine at various time points (12hrs, 24hrs, 48hrs). However, Qvd-oph is insufficient to provide a biologically significant rescue for NIM811 induced paraptosis.

**Supplemental Figure 11:** NIM811 treated cells escape an early stage of apoptosis (S11) T98G cells cultured with regular DMEM media with/without 1uM staurosporine or 15uM NIM811. Annexin-V and PI staining suggests that unlike staurosporine, most NIM811 treated cells did not go through an Annexin-V positive/PI negative stage.

**Supplemental Figure 12:** Apaf1 and MLKL depletion does not affect NIM811 induced paraptosis (S12A) si-Apaf1,si-MLKL or non-target control transfected U251 cells were collected 48hrs post-transfection for western blot to verify the knockdown efficiency. (S12B) Loss of Apaf1 or MLKL does not rescue cells from NIM811 induced paraptosis. Scale bar=50μm

**Supplemental Figure 13:** NIM811 causes cytoplasmic vacuolization in A549 and MDA-MB231 cells (S13A-13B) NIM811 induced paraptosis in A549 cells after 48hr 15μM NIM811 treatment and in MDA-MB231 cells after 32hr 10μM NIM811 treatment. S13A scale bar=50μm, and S13B scale bar=25μm.

**Supplementary Table 1**: Representative gene changes in 20hrs 10uM NIM811 treated U251 cells. NIM811 activated up-regulation of genes encode for heat shock proteins (HSPA1B, HSPB8, HSP90AA1, HSPA5, HSPA13, HSPA8, HSPA4L, HSP90AB1, HSPD1) and proteins involved in UPR (ATF3, DDIT4, DDIT3, ATF4, HSPA5) and mTOR-autophagy (GABARAPL1, MAP1LC3B, SQSTM1, EIF4EBP1, ULK1).

| **Gene Symbol** | **Protein Name** | **Fold Changes** | **Ranking** | **P-value** |
| --- | --- | --- | --- | --- |
| HSPA1B | Hsp70-1B | 40.62 | 5 | 2.54E-299 |
| HSPB8 | Hsp-beta8 | 9.00 | 65 | 6.19E-260 |
| HSPH1 | Hsp105 | 5.81 | 154 | 2.69E-123 |
| HSP90AA1 | Hsp90-alpha | 4.14 | 328 | 8.56E-65 |
| HSPA13 | Hsp70 member 13 | 3.40 | 515 | 4.03E-113 |
| HSPA8 | Hsp71 | 3.33 | 542 | 1.69E-63 |
| HSPA4L | Hsp70-4L | 3.02 | 701 | 3.62E-48 |
| HSP90AB1 | Hsp90-beta | 3.02 | 709 | 1.39E-64 |
| HSPD1 | Hsp60 | 3.00 | 719 | 1.43E-58 |
| ATF3 | ATF3 | 10.97 | 43 | 1.63E-253 |
| DDIT4 | DDIT4 | 6.73 | 114 | 4.39E-171 |
| DDIT3 | CHOP | 6.38 | 124 | 7.55E-185 |
| ATF4 | ATF4 | 4.90 | 217 | 3.74E-171 |
| HSPA5 | Bip | 3.78 | 411 | 3.07E-85 |
| GABARAPL1 | ATG8 | 21.96 | 13 | 0 |
| MAP1LC3B | LC3B | 5.08 | 196 | 5.10E-134 |
| SQSTM1 | P62 | 4.59 | 254 | 1.74E-107 |
| EIF4EBP1 | 4EBP1 | 4.08 | 339 | 3.74E-155 |
| ULK1 | ULK1 | 2.83 | 826 | 4.26E-53 |

**Supplementary Table 2**: Representative gene changes in U251 cells pre-treated with 2hrs 20uM Cycloheximide followed by 20hrs 10uM NIM811 incubation. Comparing with the NIM811 only treated cells, RNA-seq result indicates that the genes encode for heat shock proteins (HSPA1A, HSPA1B, HSP90AB3P, HSPB8, HSP90AA1, HSPH1, HSPA4L), autophagy (GABARAPL1, ULK1, SQSTM1) and UPR (DDIT4, ATF3) were significantly down regulated in cells with 2hrs cycloheximide brief treatment.

| **Gene Symbol** | **Protein Name** | **Fold Changes** | **Ranking** | **P-value** |
| --- | --- | --- | --- | --- |
| GABARAPL1 | ATG8 | -21.99 | 1 | 1.47E-57 |
| ULK1 | ULK1 | -6.38 | 25 | 1.69E-38 |
| SQSTM1 | P62 | -3.15 | 212 | 1.46E-21 |
| DDIT4 | ATF4 | -4.56 | 69 | 9.86E-38 |
| ATF3 | ATF3 | -5.85 | 31 | 4.13E-30 |
| HSPA1A | HSP701A | -12.97 | 5 | 8.46E-25 |
| HSPA1B | HSP701B | -9.71 | 8 | 4.19E-09 |
| HSP90AB3P | HSP90BC | -4.86 | 54 | 2.62E-23 |
| HSPB8 | HSP22 | -4.28 | 80 | 1.20E-30 |
| HSP90AA1 | HSP90A1 | -4.09 | 95 | 1.10E-28 |
| HSPH1 | HSP110 | -3.91 | 105 | 1.04E-25 |
| HSPA4L | HSP704L | -3.49 | 146 | 2.94E-24 |
