# Supplementary figures and images for: Cycloheximide promotes paraptosis induced by inhibition of cyclophilins in glioblastoma multiforme
Source: Cell Death Dis. 2017 May 18;8(5):e2807–. doi: 10.1038/cddis.2017.217 (PMC5520731; doi:10.1038/cddis.2017.217)

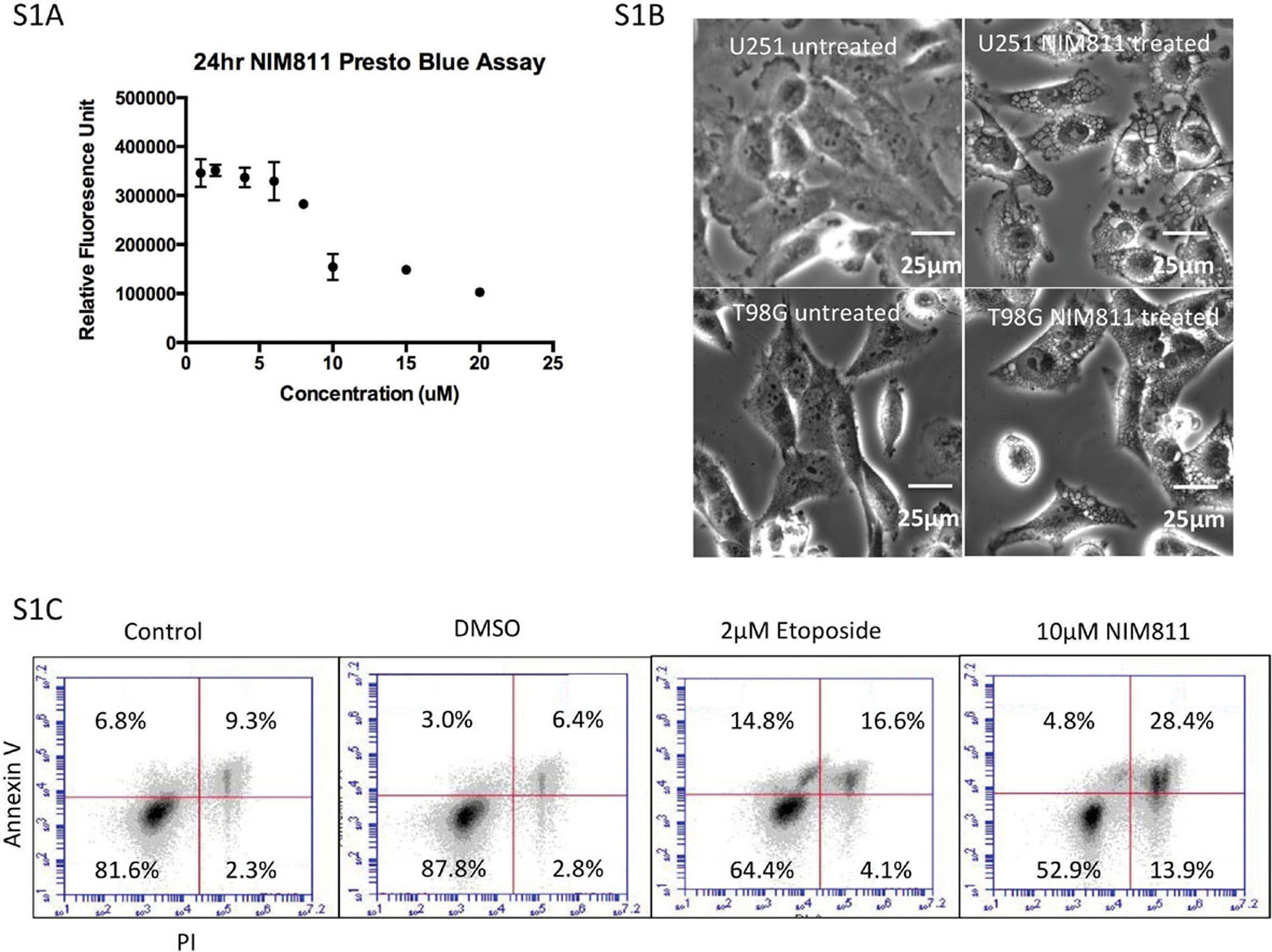

Supplement: Supplementary Figure 1 [file cddis2017217x2.tif]

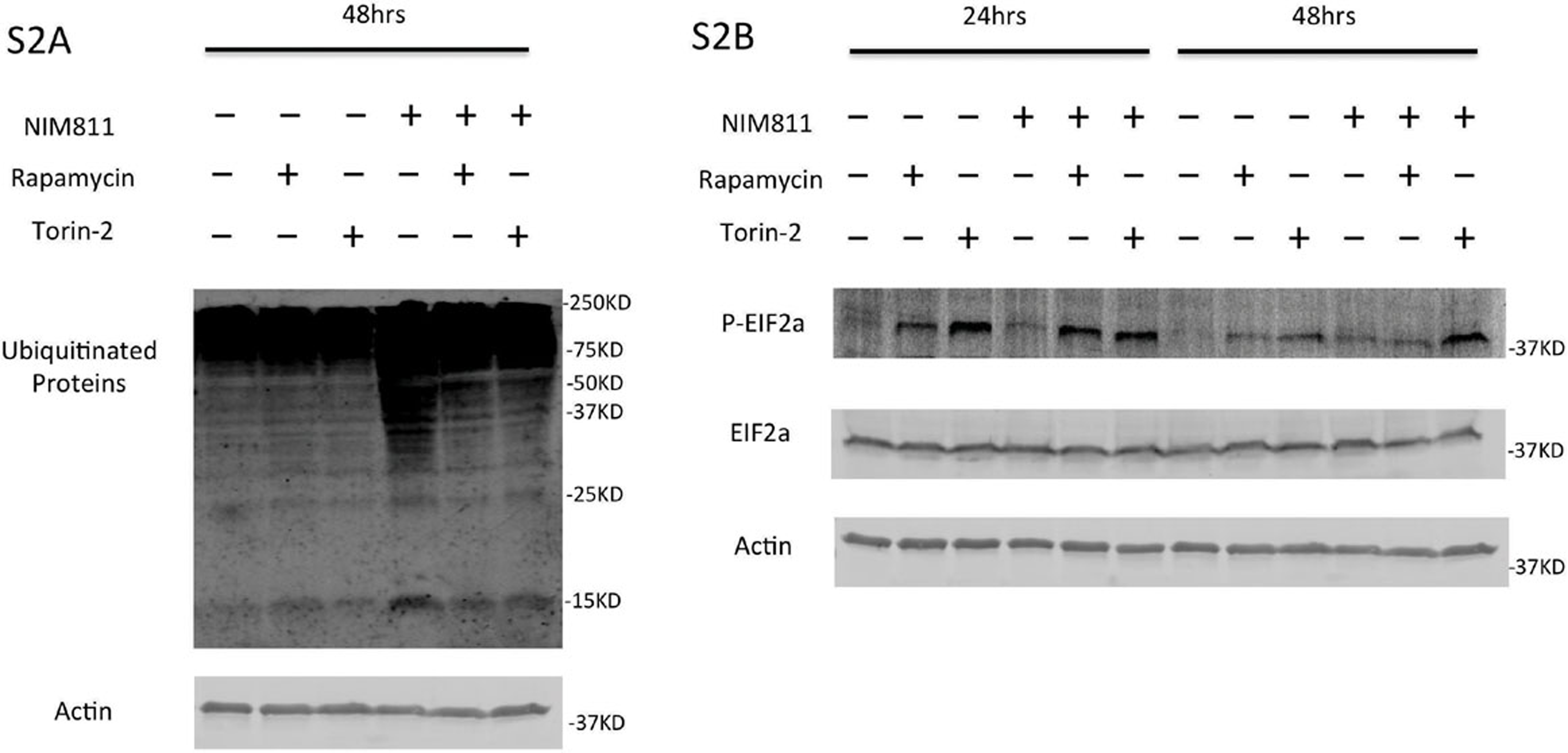

Supplement: Supplementary Figure 2 [file cddis2017217x3.tif]

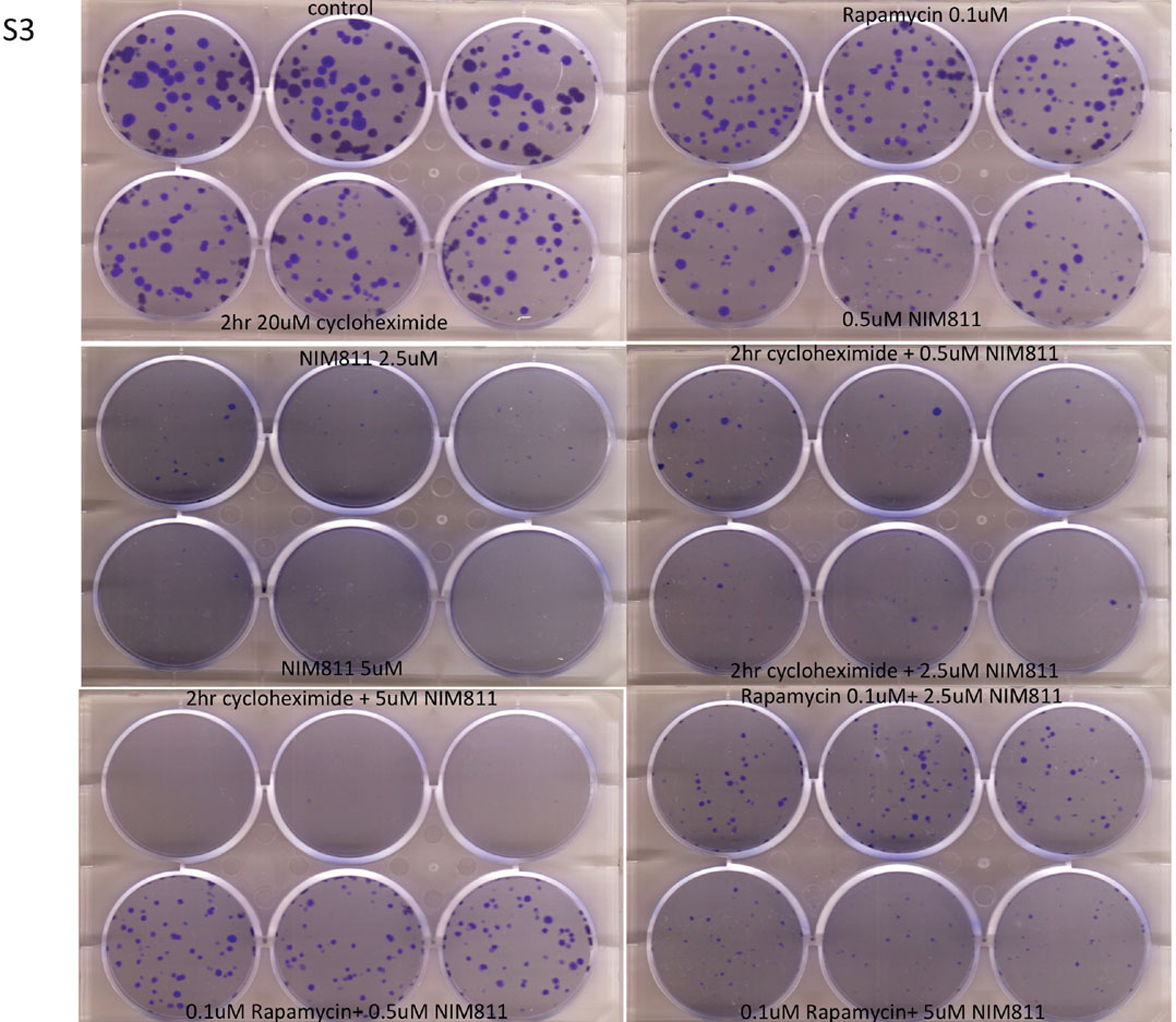

Supplement: Supplementary Figure 3 [file cddis2017217x4.tif]

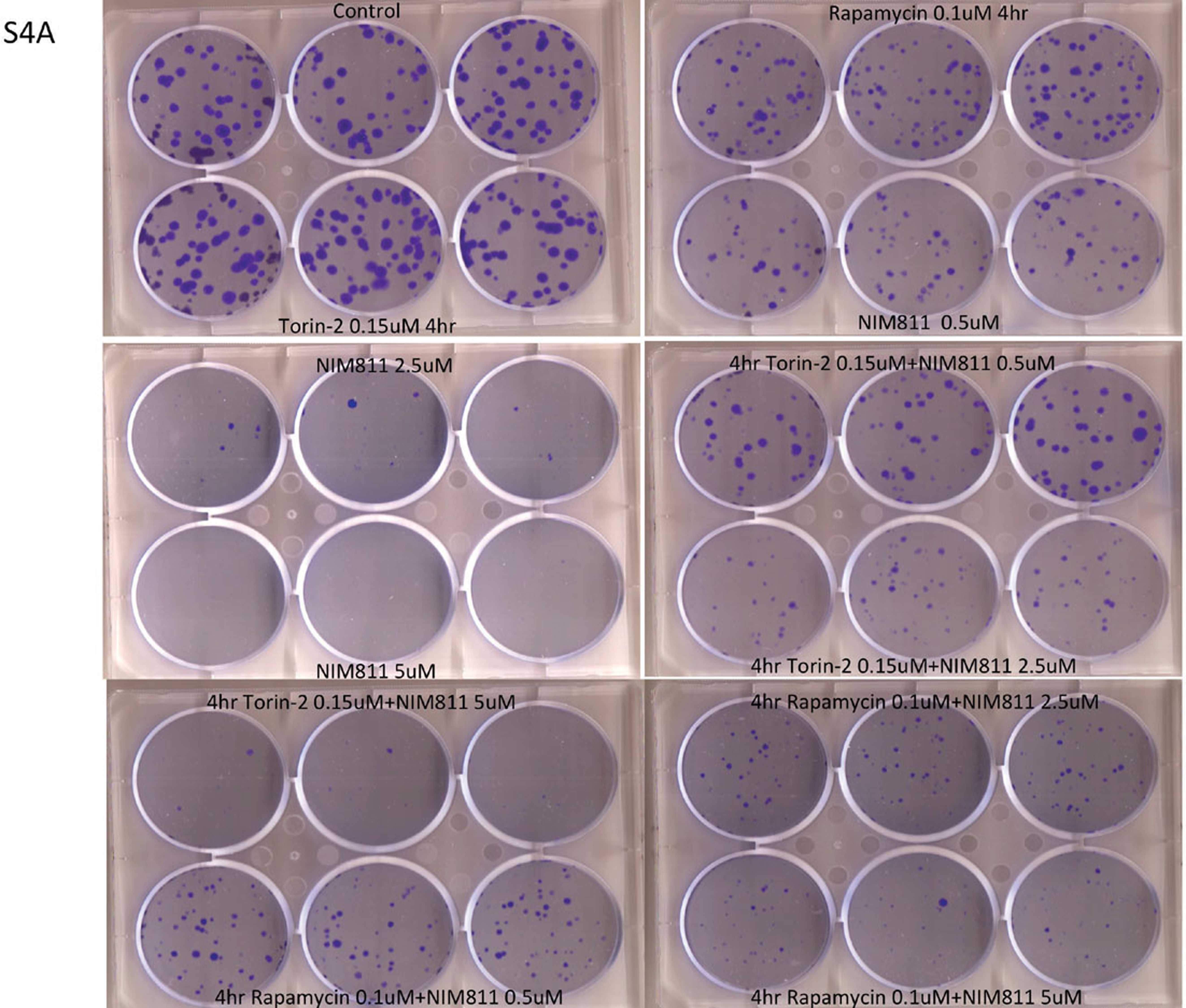

Supplement: Supplementary Figure 4 [file cddis2017217x5.tif]

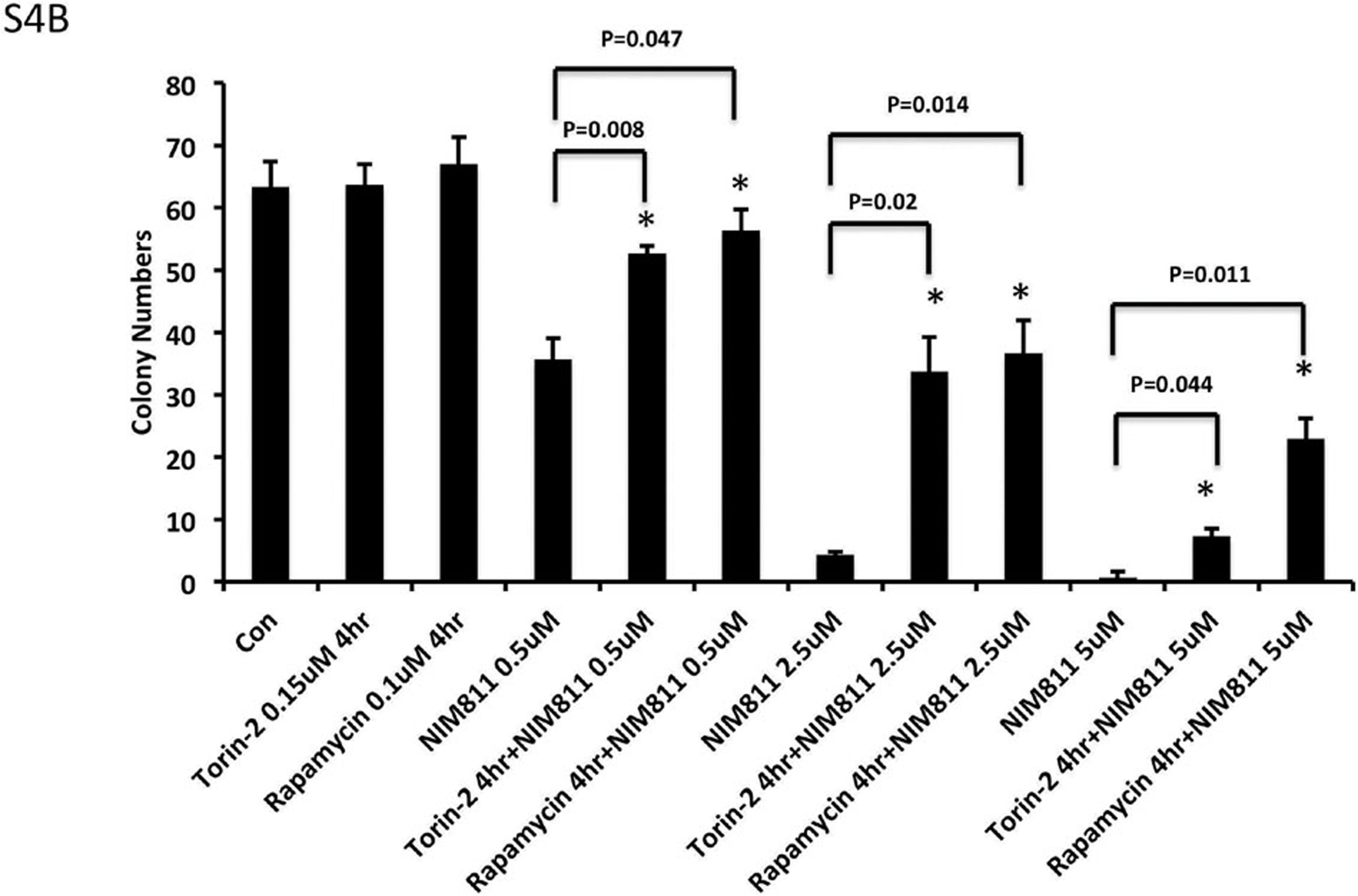

Supplement: Supplementary Figure 4A [file cddis2017217x6.tif]

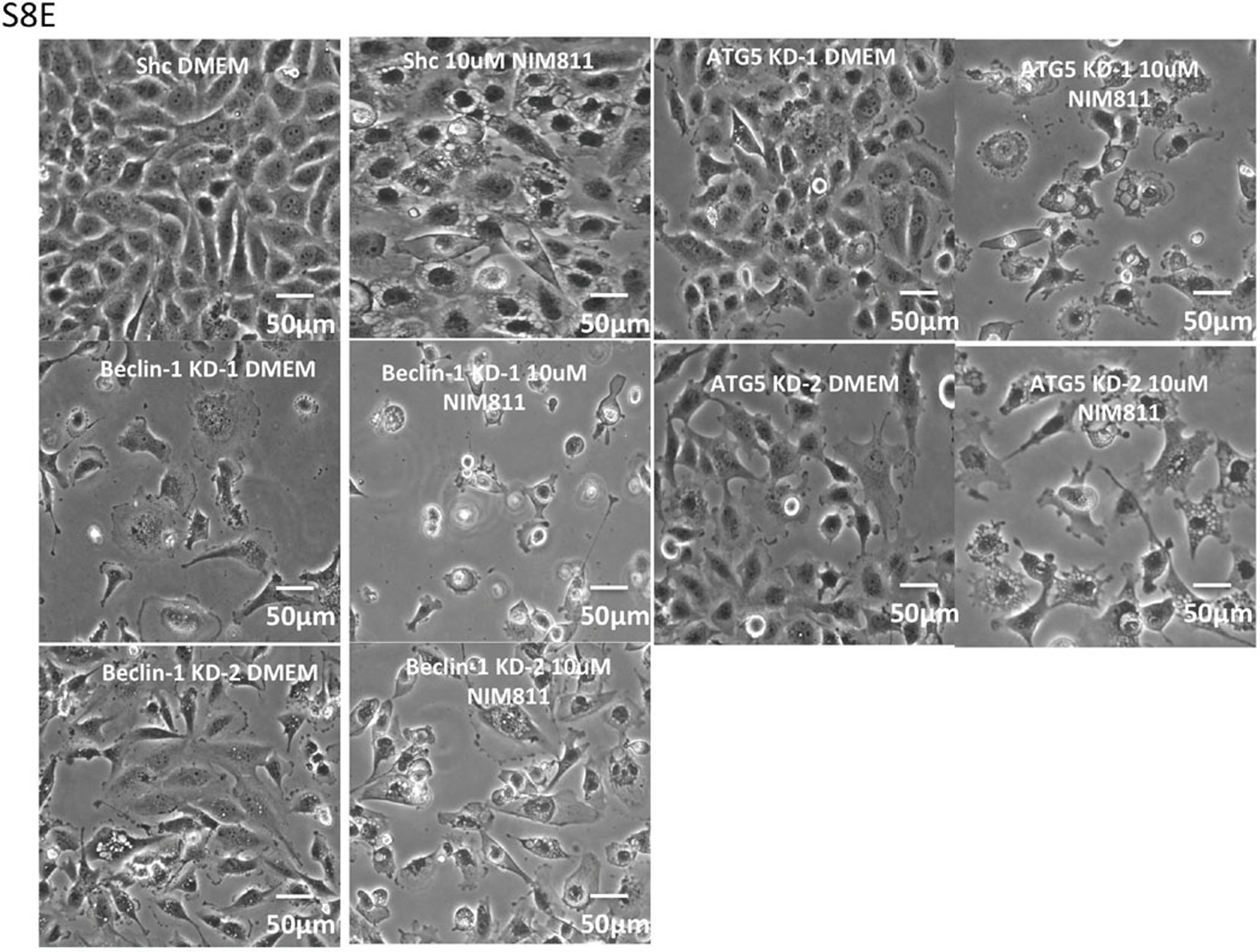

Supplement: Supplementary Figure 8A-8D [file cddis2017217x15.tif]

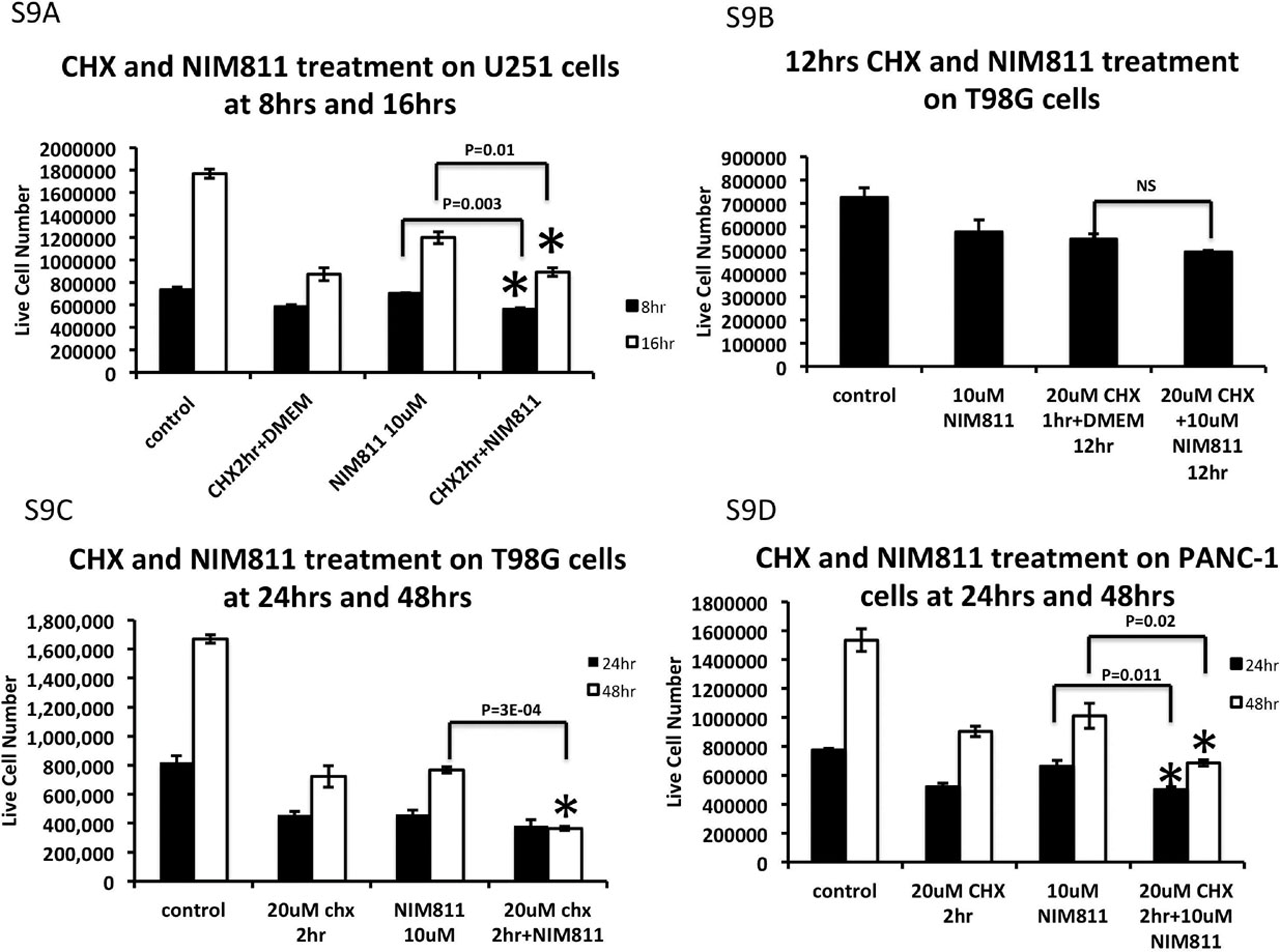

Supplement: Supplementary Figure 8E [file cddis2017217x16.tif]

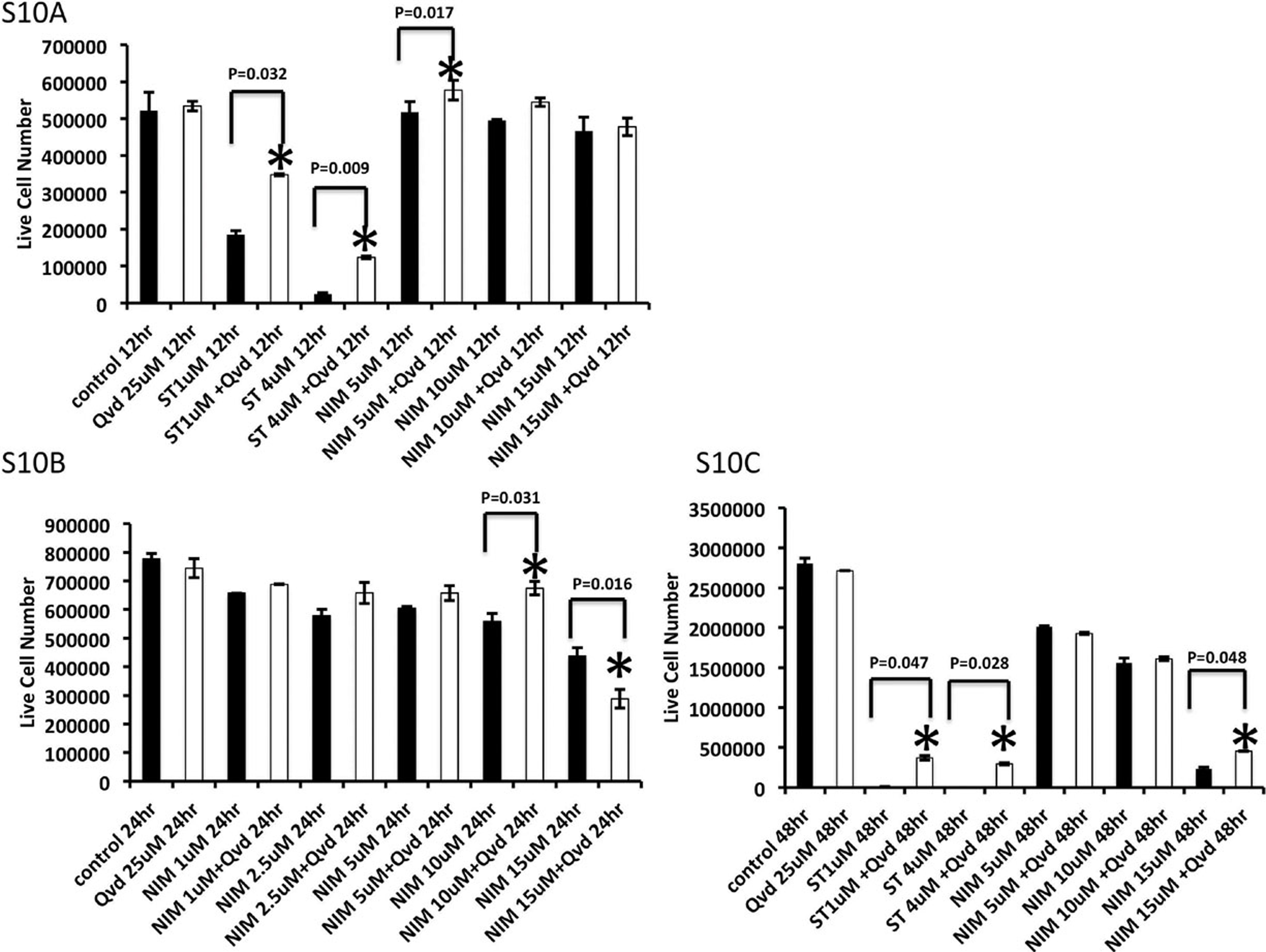

Supplement: Supplementary Figure 9 [file cddis2017217x17.tif]

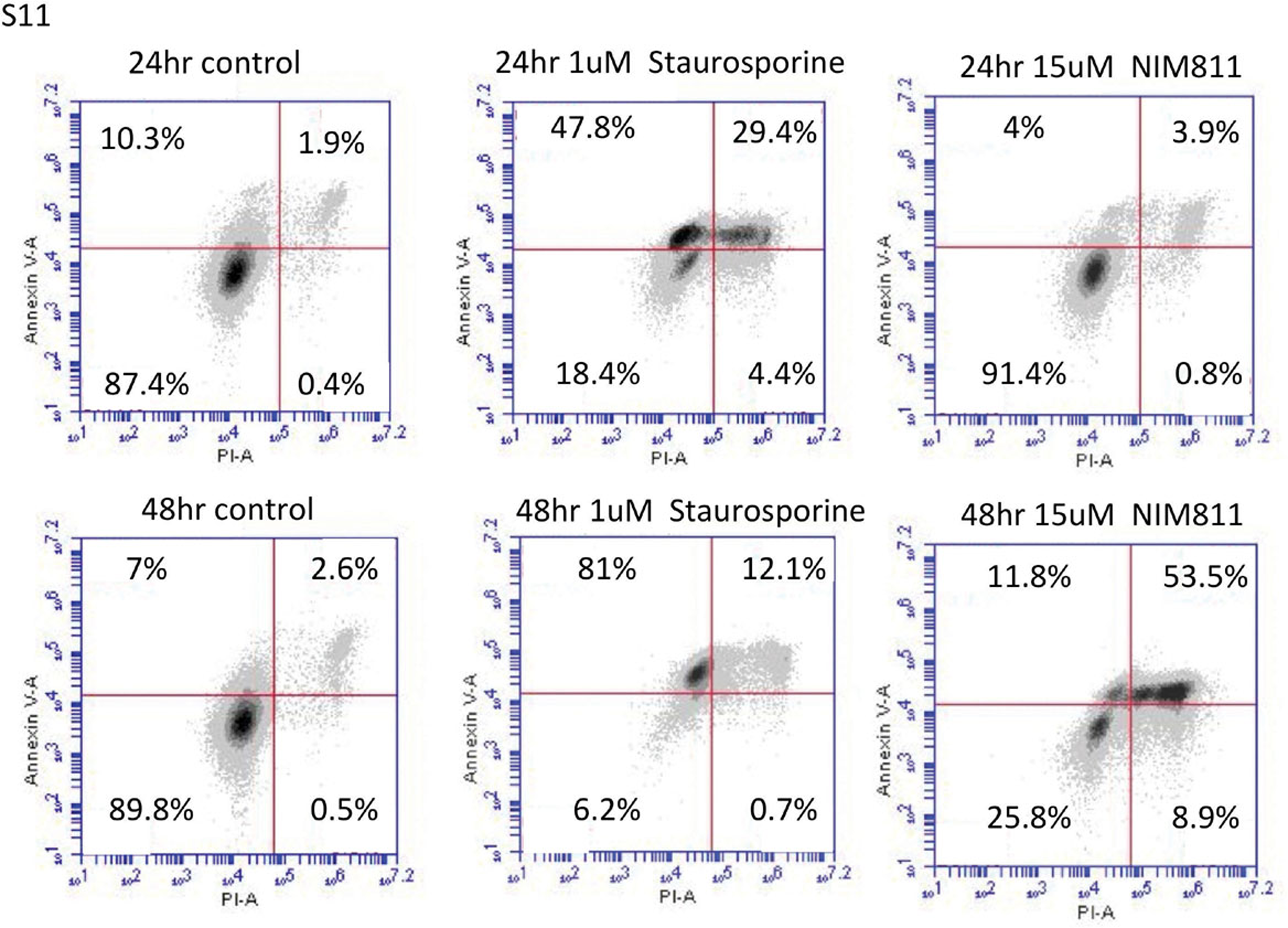

Supplement: Supplementary Figure 10 [file cddis2017217x18.tif]

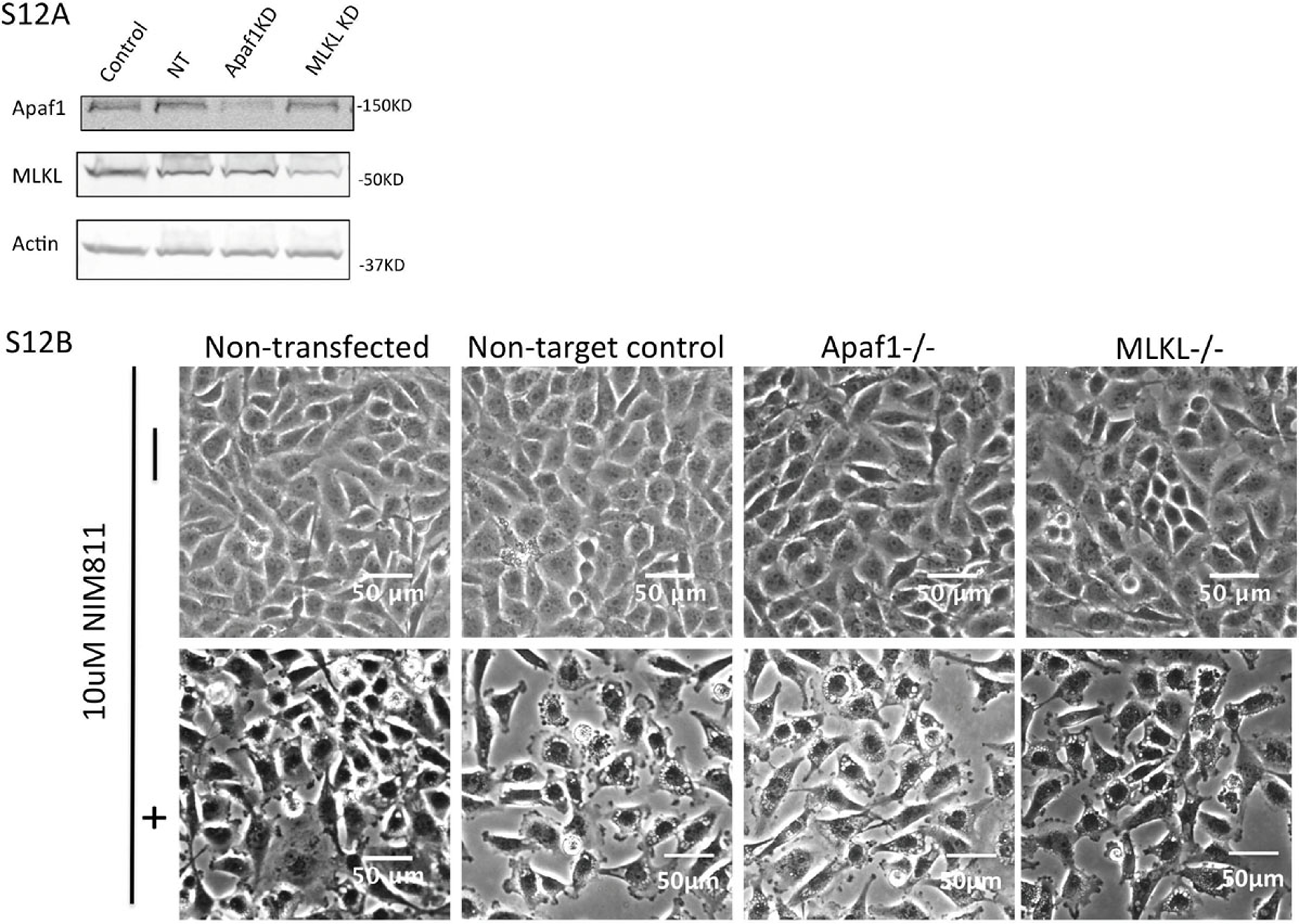

Supplement: Supplementary Figure 11 [file cddis2017217x19.tif]

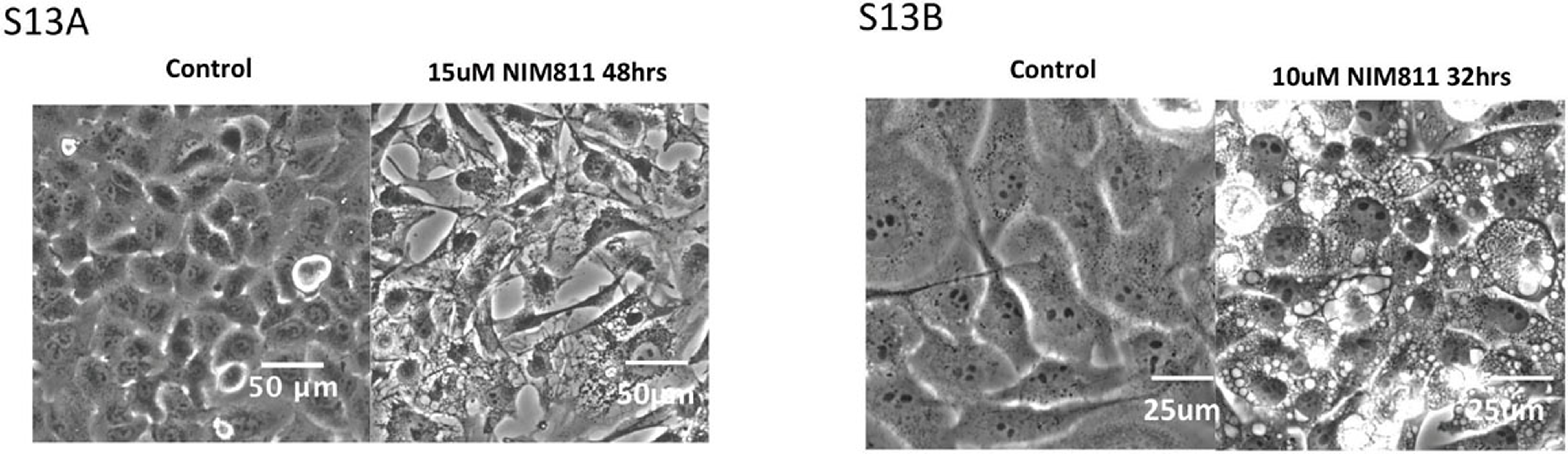

Supplement: Supplementary Figure 12 [file cddis2017217x20.tif]
